# Evaluation of targeted and high-resolution mass spectrometry methods for environmental monitoring of pharmaceuticals

**DOI:** 10.1016/j.mex.2025.103666

**Published:** 2025-10-05

**Authors:** Oksana Golovko, Mattias Sörengård, Olga Koba Ucun, Ganna Fedorova

**Affiliations:** a University of South Bohemia in České Budějovice, Faculty of Fisheries and Protection of Waters, South Bohemian Research Center of Aquaculture and Biodiversity of Hydrocenoses, Zatisi 728/II, 389 25 Vodnany, Czech Republic; b Department of Aquatic Sciences and Assessment, Swedish University of Agricultural Sciences (SLU), SE-75007 Uppsala, Sweden

**Keywords:** Pharmaceuticals, Mass spectrometry, Water analysis, Matrix effects, Wastewater

## Abstract

This study presents a comparison of three mass spectrometry-based analytical approaches—targeted tandem mass spectrometry (MS/MS), high-resolution full scan (HRFS), and data-independent acquisition (DIA)—for the quantification and screening of 74 pharmaceuticals across four environmental water matrices: tap water, river water, and influent and effluent wastewater. The methods were validated in terms of limits of quantification (LOQ), trueness, precision, and matrix effects. MS/MS exhibited the best overall performance, achieving the lowest LOQs (median 0.54 ng/L), highest trueness (median 101 %), and minimal matrix effects, confirming its suitability for routine regulatory monitoring. HRFS and DIA, while showing higher LOQs and variability, provided broader screening capabilities with acceptable trueness for 63 % and 81 % of compounds, respectively, and enabled retrospective data analysis. The methods were applied to real samples from the Živný Stream in the Czech Republic to determine pharmaceutical contamination downstream of a wastewater treatment plant (WWTP).

## Abstract (author highlights)

-A method comparison was performed using MS/MS, HRFS, and DIA for 74 pharmaceuticals.
-Matrix effects were compound- and matrix-specific, with limited predictive power for trueness.
-The study supports a complementary role of targeted and full-scan MS in environmental monitoring.

## Graphical abstract

**Figure:**
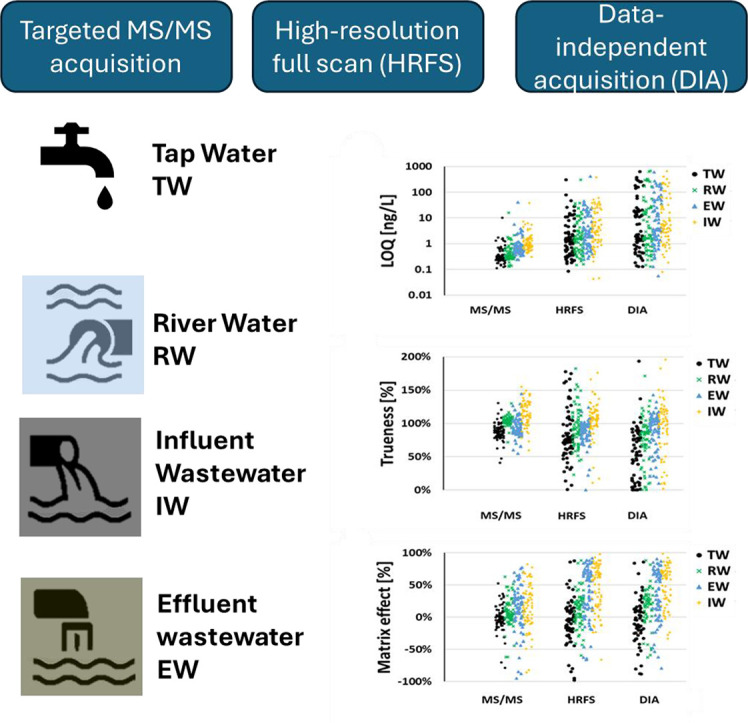

Specifications table

**Table:** 

Subject area	Environmental Science
**More specific subject area**	Environmental Analytical Chemistry – Organic Contaminants
**Name of your method**	Pharmaceuticals targeted method for four environmental matrices: tap water (TW), river water (RW), influent wastewater (IW), and effluent wastewater (EW).
**Name and reference of original method**	This analytical method is based on the method described by: **Khan, G.A., et al.,** The development and application of a system for simultaneously determining anti-infectives and nasal decongestants using on-line solid-phase extraction and liquid chromatography-tandem mass spectrometry. Journal of Pharmaceutical and Biomedical Analysis, 2012. 66: p. 24–32. doi.org/10.1016/j.jpba.2012.02.011
**Resource availability**	Not applicable

## Background

Aquatic environments are increasingly contaminated by chemicals of emerging concern (CECs) including pharmaceuticals, personal care products, illicit drugs, and perfluoroalkyl substances [1,2]. These substances typically enter wastewater systems through human excretion or improper disposal. Conventional wastewater treatment plants (WWTPs) are often ineffective at fully removing these compounds, resulting in their discharge into rivers and streams, where they may pose ecological risks [3,4]. Advancements in analytical chemistry, particularly liquid chromatography coupled with mass spectrometry (LC-MS), have significantly enhanced the detection of trace levels of CECs in complex environmental samples. High-resolution and tandem mass spectrometry (HRMS, LC-MS/MS) now enable the detection of pollutants at ng/L concentrations in various environmental matrices [5,6]. These technologies support multi-residue analyses, improving efficiency and data quality while reducing both time and costs. However, as LC-MS technologies rapidly evolve, continuous evaluation is essential to ensure reliability in detection coverage, quantification accuracy, and matrix interference. Triple quadrupole (QqQ) mass spectrometers remain the gold standard for targeted pharmaceutical analysis, offering excellent sensitivity, specificity, and robustness. Recent QqQ models feature faster data acquisition rates and the ability to monitor a greater number of analyte transitions per run. Meanwhile, Orbitrap-based instruments which offer high-resolution full-scan capabilities, are increasingly used in environmental analysis. With high resolution and accuracy, they are especially suitable for detecting a broad range of known and unknown pollutants [[7], [8], [9], [10]]. Orbitrap instruments also support advanced acquisition strategies, such as data-independent acquisition (DIA), where all precursor ions are fragmented simultaneously without prior selection. DIA provides extensive fragmentation data across wide mass ranges in a single injection, making it a powerful tool for screening and identifying unknown compounds. Moreover, DIA facilitates retrospective data analysis. However, interpreting DIA data can be complex due to overlapping signals from co-eluting compounds and difficulties in isolating clean spectra for individual analytes.

This study aimed to develop and validate an analytical method for quantifying 74 pharmaceuticals using a QqQ mass spectrometer (Quantiva Triple-Stage Quadrupole) across four environmental matrices: tap water (TW), river water (RW), influent wastewater (IW), and effluent wastewater (EW). Furthermore, the performance of targeted MS/MS acquisition using QqQ was compared with high-resolution full scan (HRFS) and DIA acquisition modes using Hybrid Quadrupole-Orbitrap (Q Exactive™). The validated methods were applied in a case study of the Živný stream, a tributary of the Blanice River in southern Czech Republic, to assess pharmaceutical contamination. The results provide insights into the strengths and limitations of different analytical platforms and inform the selection of appropriate tools for routine environmental monitoring of pharmaceuticals.

## Method details

### Materials and methods

#### Chemicals, reagents and materials

All solvents used—methanol (MeOH), acetonitrile (AcN), and isopropanol (LiChrosolv® Hypergrade)—were obtained from Merck (Darmstadt, Germany). Formic acid (LC/MS grade), used to acidify the mobile phases, was purchased from Labicom (Olomouc, Czech Republic). Ultra-pure water was produced using an Aqua-MAX-Ultra system (Younglin, Kyounggi-do, Korea). A total of 74 pharmaceutical analytical standards and corresponding mass-labeled internal standards (ISs), all of high purity (>98 %), were used. Detailed information on the individual standards is provided in the Supplementary Materials, Table SM-1. Stock spiking solutions of target analytes and ISs were prepared in MeOH at concentrations of 1 μg/mL and stored at –20 °C until use.

#### Instrumentation

The method was adapted from Khan et al. (2012) [11] which established online SPE-LC–MS/MS workflows for antibiotics and nasal decongestants in treated sewage effluent and surface water. In this study, several modifications were implemented, including the use of Accucore aQ columns, updated instrumentation (TSQ Quantiva and Q Exactive Orbitrap), the inclusion of HRFS and DIA acquisition modes, and validation across an expanded list of 74 compounds in four matrices. These adaptations extend the applicability of the original protocols and enable direct comparison between targeted and full-scan acquisition approaches.

##### Liquid chromatography

Chromatographic separation was carried out using an Accela 1250 LC pump and an Accela 600 LC pump (Thermo Fisher Scientific, San Jose, CA, USA), connected to an HTS XT-CTC autosampler (CTC Analytics AG, Zwingen, Switzerland).

The extraction column was an Accucore aQ (10 mm × 2.1 mm i.d., 3 μm particle size), and the analytical column was an Accucore aQ (50 mm × 2.1 mm i.d., 2.6 μm particle size), both from Thermo Fisher Scientific. Chromatographic parameters are listed in Supplementary Table SM-2.

The extraction and analysis were completed in 13 min using 1 mL of sample. Mobile phases were Milli-Q water with 0.1 % formic acid (A) and acetonitrile with 0.1 % formic acid (B). The flow rate was 0.3 mL/min. For the analytical pump, the gradient was 5 % B (1 min) to 100 % B (8 min), held to 10 min, re-equilibrated at 10.1 min, and maintained to 13 min. For the high-flow pump (on-line extraction), the gradient was 0 % B to 100 % B (1.07 min), held to 10 min, re-equilibrated at 10.1 min, and maintained to 13 min. Gradient details are provided in Table SM2.

##### Mass spectrometry - triple quadrupole

A TSQ Quantiva triple-stage quadrupole mass spectrometer (Thermo Fisher Scientific) was used for targeted MS/MS detection of the 74 pharmaceuticals and their metabolites. Ionization was performed using heated electrospray ionization (H-ESI) in positive mode ([*M* + *H*]^+^, with a spray voltage of 3500 V. Nitrogen (purity >99.999 %) was used as sheath gas (42 AU), auxiliary gas (12 AU), and sweep gas (1 AU). The vaporizer and capillary temperatures were set to 338 °C.

Two selected reaction monitoring (SRM) transitions were monitored per analyte. Data were processed using TraceFinder™ 3.3 software (Thermo Fisher Scientific). MS/MS acquisition parameters are summarized in Supplementary Table SM-1.

##### Mass spectrometry - orbitrap

High-resolution full scan (HRFS) and data-independent acquisition (DIA) analyses were conducted on a Q Exactive™ Hybrid Quadrupole-Orbitrap mass spectrometer (Thermo Fisher Scientific). Instrument calibration was performed daily using a calibration mixture (Thermo Fisher Scientific). Ionization was achieved using H-ESI in positive mode ([*M* + *H*]^+^), with a spray voltage of 3500 V. Nitrogen was used as sheath gas (40 AU) and auxiliary gas (10 AU). The vaporizer and capillary temperatures were set to 270 °C and 250 °C, respectively.

HRFS Mode: Full-scan spectra were acquired with a resolving power of 70,000 FWHM across an *m/z* range of 100–1000. AGC target was set at 3e6, injection time at 100 ms, and scan rate at 1 scan/s.

DIA Mode: Two scan events were used:

1. scan event: isolation width of 100 Da, resolving power 17,500 FWHM, AGC target 1e6, injection time 50 ms, loop count 4, MSX count 1, with isolation window 110 *m/z*. Stepped normalized collision energy (NCE) was set at 30 and 80.
2. scan event: isolation width of 500 Da. Most of parameters were the same except of some of them; loop count 1, with isolation window 510 *m/z* and inclusion list centered at *m/z* values of 150, 250, 350, 450, and 750.

A mass tolerance of 10 ppm was applied. Data were processed using TraceFinder™ 3.3. Detailed instrument parameters are listed in Supplementary Table SM-1.

#### Sample collection

For method validation, influent and effluent wastewater samples were collected from the municipal WWTP in České Budějovice, Czech Republic. The WWTP employs a biological activated sludge process with partial nitrification and thermophilic anaerobic sludge stabilization. It has a capacity of 90,000 m³/day and serves approximately 112,000 inhabitants. The influent primarily consists of municipal wastewater, with industrial contributions below 5 %. The facility also receives input from the České Budějovice Regional Hospital.

Twenty-four-hour time-proportional (15 min intervals) composite samples were collected using an automated sampler (ASP-STATION 2000, *E* + *H*). All samples were collected in high-density polyethylene bottles, immediately frozen, and stored until analysis. Tap water was collected from the laboratory facilities of the Faculty of Fisheries and Protection of Waters, South Bohemian Research Center of Aquaculture and Biodiversity of Hydrocenoses, located in Vodňany, Czech Republic. Surface water samples for method validation were collected from the Blanice River near Vodňany. For method application, additional surface water samples were collected from the Živný Stream (ZS), a tributary of the Blanice River, during both spring and autumn. Four sampling sites were selected:

C: Control site, 4.5 km upstream of the Prachatice WWTP (representing background conditions),
E: 100 m downstream of the WWTP discharge point,
U: 1.5 km downstream of the WWTP discharge,
R: 3 km downstream of the WWTP discharge.

#### Sample preparation

Seven replicate 10 mL aliquots of thawed wastewater and surface water samples were filtered through 0.45 μm regenerated cellulose syringe filters (Labicom, Olomouc, Czech Republic). Samples—including influent (IW), effluent (EW), river water (RW), tap water (TW), and Živný Stream samples (ZS)—were spiked with the IS mixture at 50 ng/L. Samples were processed using an online solid-phase extraction coupled with liquid chromatography (on-line SPE-LC) method [11,12].

#### Statistical analysis

Student’s *t*-test was used for pairwise comparisons of LOQ values, as it is widely applied in analytical validation studies and provides a straightforward means of testing differences between acquisition modes. Kendall’s tau was selected for correlation analyses of trueness and matrix effects because it is non-parametric and robust to non-normal distributions and outliers, which are common in environmental data. Spearman’s rank correlation was used in the case study to evaluate associations between upstream, downstream, and influent concentrations; given the exploratory nature of this analysis, no correction for multiple comparisons was applied. All statistical analyses were performed using Microsoft Excel (Microsoft Corp., Redmond, WA, USA).

## Method validation

The performance of the three methods (MS/MS, HRFS, and DIA) was evaluated in terms of linearity, limits of quantification (LOQs), trueness, precision, and matrix effects across four matrices: TW, RW, IW, and EW.

Calibration curves were prepared in ultrapure water across concentration ranges of 1–1000 ng/L (RW and TW) and 1–10,000 ng/L (IW and EW). Instrument stability was monitored by measuring calibration curves at the beginning and end of each sequence. LOQs were defined as $\frac{1}{4}$ of the lowest calibration point with a relative standard deviation (RSD) of the average response factor <30 %. Individual LOQs were determined based on signal intensity at this concentration.

Trueness was assessed by spiking samples at the following concentration levels:

TW: 10 and 100 ng/L
RW: 50 and 500 ng/L
IW and EW: 500, 1000, and 5000 ng/L
  (1) $$\text{Trueness}\;\left(\%\right)=\frac{\left(Peak\;area\;\left(pre-spiked\right)-Peak\;area\;\left(Blank\right)\right)}{\left(Peak\;area\;\left(post-spiked\right)-Peak\;area\;\left(Blank\right)\right)}\times 100$$

Matrix effects were corrected using matrix-matched calibration standards (MST), which were prepared by spiking ISs at 50 ng/L and native analytes at 5000 ng/L (IW and EW), 100 ng/L (RW), and 50 ng/L (TW). The matrix-affected response factor was calculated as the difference between the peak area-to-IS ratio in matrix-matched standards and non-spiked samples.

(2) $$\text{Matrix}\;\text{effects}\left(\%\right)=\frac{\left(Peak\;area\;MST-Peak\;area\;Blank\right)}{Peak\;area\;Solvent}\times 100$$

Precision was evaluated as the RSD of the analyte concentrations measured in seven replicates. Tap water (*n* = 3) and ultrapure water (*n* = 3) blanks were prepared and analyzed under the same conditions; no target compounds were detected.

Most target analytes were detected across all methods and matrices and showed good linearity, with R² values ranging from 0.969 to 0.9999 (see Supplementary Table SM-3).

## Limit of quantification (LOQ)

**Fig. 1:**
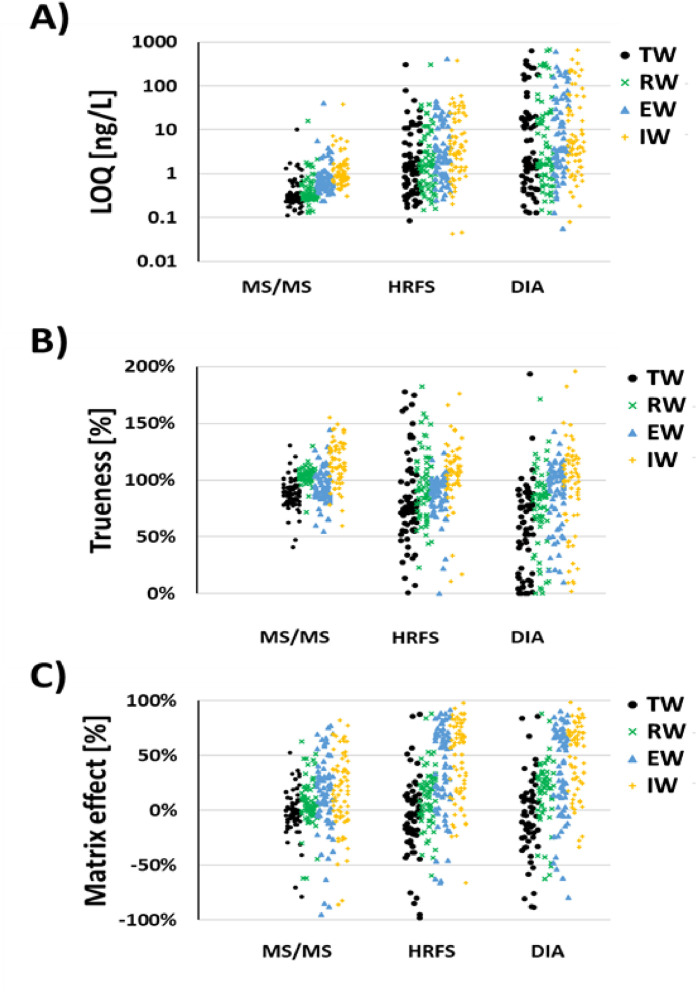
A) Limit of quantifications (LOQs) [ng/L], B) Trueness [%] and C) matrix effects assessed on 74 spiked pharmaceuticals in four different matrices: tap water (TW, black circles), river water (RW, green crosses), effluent wastewater (EW, blue triangles) and influent wastewater (IW, yellow pluses) using conventional target analysis (MS/MS), high-resolution full scan (HRFS) and data-independent acquisition (DIA) modes.

Low LOQs are essential for accurate detection of trace-level pharmaceuticals in the environment. Our results demonstrated that across all four matrices—TW, RW, EW and IW—the targeted MS/MS method consistently achieved the lowest LOQs, ranging from 0.11 to 40 ng/L, with a median of 0.54 ng/L (Fig. 1A; SM-3). HRFS exhibited LOQs up to 680 ng/L (median: 3.7 ng/L), while DIA showed a maximum of 420 ng/L (median: 1.9 ng/L), representing 6.9- and 3.5-fold increases over MS/MS, respectively.

As expected, matrix complexity significantly influenced LOQs. All three methods showed a statistically significant (*p* < 0.05, Student’s *t*-test) increase in both LOQ values and variability (Q3-Q1 range) with increasing matrix complexity. For example, comparing TW to IW, the relative median LOQ increased by 350 % (MS/MS), 270 % (HRFS), and 270 % (DIA), while relative variation (calculated as [(Q3-Q1)/median] × 100) rose by 54 %, 960 %, and 310 %, respectively. However, pharmaceutical concentrations in wastewater samples are typically high, making the use of methods with higher limits of quantification (LOQs) less critical.

Interestingly, despite generally higher LOQs, the screening methods (HRFS and DIA) achieved very low LOQs in complex matrices—down to 0.078 ng/L (Donepezil in DIA) and 0.042 ng/L (Sulfamethoxazole in HRFS) in influent wastewater.

Overall, targeted MS/MS clearly outperformed both screening approaches in terms of low and consistent LOQs across all matrices. This supports its continued use for routine monitoring of known contaminants at trace levels. However, HRFS and DIA serve complementary roles. These full-scan methods enable broad-spectrum screening, facilitating the detection of “known unknowns” and “unknown unknowns” [10]. While identification confidence depends on spectral accuracy and database matching, high-resolution mass spectrometry offers a distinct advantage over DIA in distinguishing compounds with similar *m/z* values, thus minimizing false positives.

A practical benefit of DIA is that this method can be conducted on the same instrument platform as MS/MS (e.g., QqQ systems), allowing efficient integration into existing workflows. Although DIA lacks the mass resolution of HRFS, both methods benefit from post-acquisition data mining, enabling retrospective analysis of stored MS/MS data without re-analysis [9].

The present study was designed to compare quantitative performance across two instruments (QqQ and Orbitrap) and three acquisition modes (MS/MS, DIA, HRFS). While MS/MS unsurprisingly provided the lowest LOQs and most consistent trueness, our results show that both DIA and HRFS also yielded reliable quantitative data for the 74 target compounds. This is important because, although MS/MS remains the gold standard for trace-level quantification, full-scan methods such as HRFS and DIA can also serve in a quantitative role, with the added benefit that their data can be retrospectively mined for additional analytes. In this work we did not perform suspect or non-target screening; therefore, our conclusions regarding HRFS and DIA are limited to their demonstrated quantitative capabilities.

## Trueness and precision

In the next validation step, the trueness of each method—commonly referred to as recovery—was evaluated by comparing measured concentrations against theoretical spike levels. Trueness values were calculated using Eq. (1) and are summarized in Table SM-4 and visualized in Fig. 1B.

Trueness values within the 50–150 % range are generally considered acceptable for environmental analysis [13]. The targeted MS/MS method achieved the highest overall trueness, with 96 % of compounds falling within the acceptable range and a median value of 101 %. DIA and HRFS performed slightly lower, with 81 % and 63 % of compounds, respectively, within the acceptable range. Their corresponding median trueness values were 94 % (DIA) and 86 % (HRFS).

Interestingly, the best trueness was not consistently observed in the cleanest matrix (TW). Instead, optimal performance occurred in RW for MS/MS, and in IW for both DIA and HRFS. This could be due to matrix-specific interactions or enhanced signal recovery in the presence of certain co-extractives [14].

As with LOQs, trueness variation increased with matrix complexity. DIA showed particularly low trueness in TW, with 31 % of compounds falling below the 50 % threshold. This reduction may result from signal suppression due to co-eluting matrix constituents at similar *m/z* values, particularly problematic for DIA given its broader fragmentation and lower mass resolution. HRMS offers an advantage here by more effectively separating compounds with overlapping masses, improving selectivity and reducing potential false positives.

Fig. 1B illustrates greater trueness variability in TW and RW for HRFS compared to other matrices. While most compounds still met the 50–150 % threshold, a notable cluster of low-trueness outliers was observed for DIA in TW and RW, with several compounds exhibiting recoveries between 0 and 20 %.

**Fig. 2:**
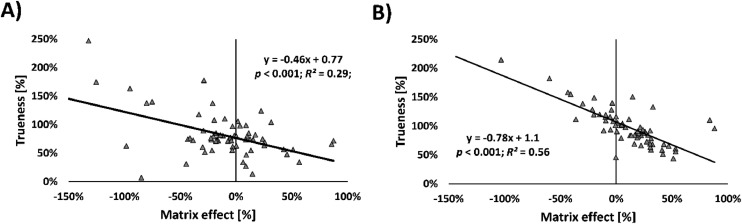
Correlation between matrix effects [%] and trueness [%] for high-resolution full scan (HRFS) acquisition mode in A) tap water (TW) and B) river water (RW).

Correlation analysis (Fig. 2) indicated that only 27 out of 66 comparisons (41 %) yielded statistically significant positive correlations in trueness across methods and matrices (*p* < 0.001, Kendall test). Moreover, the overall correlation was weak (mean R² = 0.09), suggesting that no consistent pattern emerged in which specific compounds systematically showed high or low trueness across different analytical setups. This highlights that trueness variability is largely compound- and matrix-specific.

Precision was evaluated for all 74 pharmaceuticals across four matrices (TW, RW, IW, EW), with replicate measurements (*n* = 7) at relevant spiking levels (see Supplementary Table SM-4 for full results). Most compounds showed RSD values below 30 %, in line with standard acceptance criteria for environmental trace analysis. A few analytes exhibited higher RSDs (31–45 %) at the highest spiking level (500 ng/L) particularly in RW and IW, likely due to matrix interferences and partial saturation. These cases were rare and occurred at concentrations above those typically observed in the environment.

## Matrix effects

The effect of different matrices on target compounds was evaluated by comparing analyte responses in matrix-matched standards (MSTs) with those in calibration standards prepared in ultrapure water, using Eq. (2) (see also Fig. 1C and Supplementary Table SM-5). Matrix effects arise when co-eluting matrix components alter the ionization efficiency of analytes, either through ion suppression or enhancement. These effects can lower sensitivity and, if not adequately compensated for, negatively impact method accuracy—especially in the absence of perfectly matched internal standards. TW exhibited the lowest matrix effects, while IW showed the highest, Fig. 1C. Instrumentation and acquisition mode also had a significant impact. The targeted MS/MS method displayed the lowest matrix effect in TW, with a median of –2 % and an interquartile range (IQR) from –8 % to 6 %. In IW, the matrix effect increased to a median of 16 %. Both HRFS and DIA showed similarly low matrix effects in TW, with median values of –6 % (HRFS) and –4 % (DIA) and IQRs of –28 % to 10 % and –24 % to 12 %, respectively.

As matrix complexity increased from TW to IW, the extent and variability of matrix effects grew for all methods. However, no consistent trend was observed regarding whether ion suppression or enhancement was dominant.

Correlation analysis of matrix effects across methods and matrices (Fig. 2) showed 42 out of 66 (79 %) significant and positive associations (*p* < 0.001, Kendall’s tau). This suggests that many individual compounds were consistently prone to matrix effects—either suppression or enhancement—regardless of method or matrix. The weakest correlation was found for TW (6 of 30 significant correlations, 20 %), likely due to the inherently low levels of matrix interference in this cleaner matrix.

While matrix effects are commonly linked to reduced trueness, our analysis showed that matrix effects alone did not strongly predict trueness across all methods. The overall average R² between matrix effects and trueness was 0.14. Notably, a stronger relationship was observed for the HRFS method in TW (R² = 0.26, *p* < 0.001) and in RW (R² = 0.56, *p* < 0.001), suggesting that matrix effects may play a more pronounced role in trueness for full-scan acquisition in cleaner matrices (Fig. 2). These results indicate that in HRFS, even minor co-eluting substances may cause notable ion suppression or enhancement that impacts quantification accuracy.

Furthermore, the sensitivity of HRFS and DIA to matrix purity was evident, particularly in cleaner matrices like TW and RW, where matrix effects were unexpectedly more variable compared to MS/MS (Table SM-5, Fig. 2). For DIA, one possible explanation is the inability to isolate precursor ions prior to fragmentation. Since all ions are fragmented simultaneously in DIA, co-eluting compounds—even with very different precursor *m/z* values—can contribute to mixed product ion spectra, complicating compound identification and quantification. This limitation makes DIA more susceptible to matrix interferences.

It should be noted that the present method was restricted to positive electrospray ionization (H-ESI +). While this mode is optimal for most pharmaceuticals investigated, it is less suited to strongly acidic compounds such as diclofenac, ibuprofen and naproxen, which typically ionize more efficiently in negative mode. As a result, sensitivity for these analytes may be reduced compared to polarity-switching or dedicated H-ESI – methods. The choice of H-ESI + was made to maximize sensitivity for most analytes (>80 %) and to maintain feasible run times for high-throughput analysis. Nonetheless, for comprehensive pharmaceutical screening, the present method should be complemented with negative-mode acquisition or polarity-switching workflows, particularly when acidic pharmaceuticals are of interest.

## Case study and method application

**Fig. 3:**
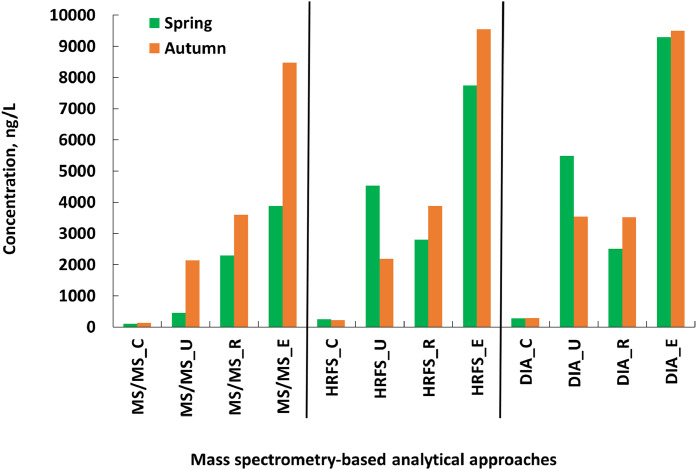
Sum of average concentrations of 74 pharmaceuticals detected in the Živný Stream in Spring and Autumn 2015 (*n* = 10).

As the next step, the validated methods were employed to determine pharmaceuticals in real environmental samples collected from the Živný Stream (ZS), a tributary of the Blanice River in southern Czech Republic to demonstrate practical applicability of the methods. Across all sampling sites and timepoints, the detection frequency of pharmaceuticals increased with proximity to the WWTP. Upstream river water (Site C) exhibited an average detection frequency of 14 %, compared to 42 % and 48 % for downstream surface water (Sites R and E), Fig. 3, Table SM-6. Caffeine showed the highest relative concentration in upstream water, reflecting its ubiquitous use and environmental persistence. In contrast, downstream sites had elevated levels of telmisartan, tramadol, valsartan, and diclofenac.

C: Control site, 4.5 km upstream of the Prachatice WWTP (representing background conditions), U: 1.5 km downstream of the WWTP discharge, R: 3 km downstream of the WWTP discharge, E: 100 m downstream of the WWTP discharge point.

**Fig. 4:**
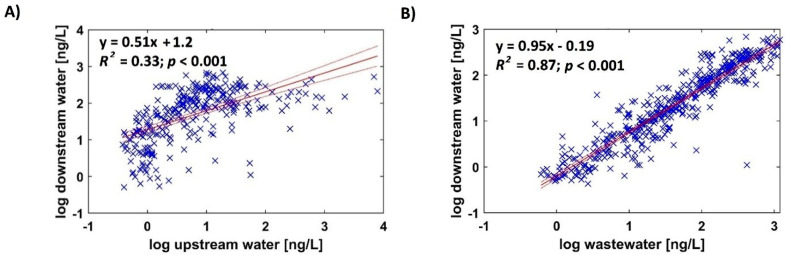
Pharmaceutical concentrations (log scale) detected in the Živný Stream downstream of a WWTP (*n* = 74) from Spring and Autumn 2015 (*n* = 10). Spearman regressions with A) upstream river water and B) WWTP influent concentrations using MS/MS.

Temporal trends were minimal, with no significant variation observed over the monitoring period (Table SM-6). However, log-transformed pharmaceutical concentrations in downstream samples correlated strongly with both upstream concentrations (Spearman’s r² = 0.33, *p* < 0.001) and influent wastewater concentrations (r² = 0.87, *p* < 0.001), Fig. 4.

Multilinear regression using both upstream and influent data yielded an (r² = 0.81), not exceeding the predictive value of the WWTP influent alone. This indicates that the WWTP is the primary source of pharmaceuticals in the downstream Živný Stream. The regression intercept (–0.19) suggests a ∼50 % dilution of WWTP effluent in the stream, Fig. 4. These findings support the characterization of WWTPs as major point sources of pharmaceuticals and underscore the importance of advanced treatment technologies for emission reduction [1,15].

Most pharmaceutical concentrations were below LOQs for all methods in the upstream control samples. Exceptions included metoprolol, metoprolol acid, tramadol, carbamazepine, 10,11-trans-dihydroxy-10,11-dihydrocarbamazepine, irbesartan, telmisartan, and caffeine, which were detectable in both spring and autumn seasons. Sulfapyridine and diclofenac were also present in autumn, while clarithromycin and valsartan appeared in spring samples.

**Fig. 5:**
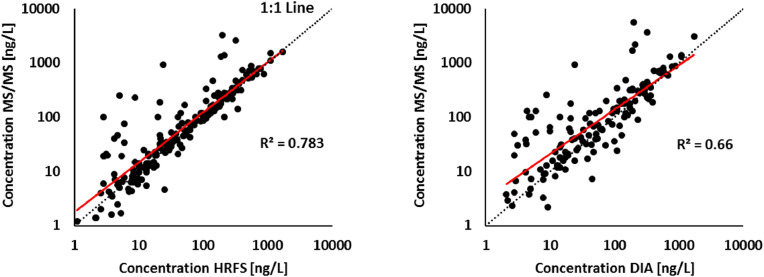
Linear correlation (red) between conventional target analysis (MS/MS) and A) high-resolution full scan (HRFS) and B) data-independent acquisition (DIA) modes assessed on detected (out of 74) pharmaceuticals in case study Živný Stream water, Czech Republic, and conflating with effluent waste water between Spring and Autumn (*n* = 10) 2015. Solid line represents the 1:1 fit.

To compare method performance in real samples, concentrations obtained via MS/MS were plotted against those from HRFS and DIA in a cross-plot (Fig. 5). MS/MS results showed strong correlations with both HRFS (R² = 0.78, *p* < 0.001) and DIA (R² = 0.66, *p* < 0.001), confirming the reliability of full-scan methods for target screening. Despite their limitations (higher LOQs, more variable trueness, and greater matrix effects), both HRFS and DIA demonstrated good agreement with targeted MS/MS.

However, deviations from the 1:1 regression line at lower concentrations suggest potential overestimation by HRFS and DIA, likely due to unresolved matrix effects or reduced selectivity at low signal intensities (Fig. 5, Table SM-6).

The Živný Stream application uses samples collected in spring and autumn 2015 and is presented as a historical baseline to demonstrate method applicability rather than to infer temporal trends. Recent Czech studies (2015–2025) continue to report a similar suite of pharmaceuticals and concentration ranges in rivers and WWTP effluents—e.g., diclofenac, telmisartan, tramadol, metoprolol, and carbamazepine in the ng/L–µg/L range in effluents and impacted tributaries; tens to hundreds of ng/L for anticonvulsants in surface waters [[16], [17], [18], [19]]. These observations support the ongoing relevance of our compound list and conclusions for effluent-impacted reaches. We also note evolving EU Watch List priorities [[20], [21], [22]], with recent emphasis on antibiotics, while legacy compounds such as diclofenac remain widely monitored. For comprehensive surveillance, updated local monitoring is recommended and our validated methods are directly transferable to contemporary samples.

## Limitations

Not applicable.

## Ethics statements

The authors have read and follow the ethical requirements for publication in MethodsX. This work meets all ethic requirements. This work does not involve studies with animals and humans.

## Related research article

None.

## CRediT author statement

**Oksana Golovko**: Conceptualization, Data curation, Formal analysis, Investigation, Methodology, Writing – original draft, **Mattias Sörengård**: Data curation, Formal analysis, Investigation, Writing – review and editing, **Olga Koba Ucun**: Conceptualization, Writing – review and editing, **Ganna Fedorova:** Conceptualization, Writing – review and editing

## Declaration of competing interest

The authors declare that they have no known competing financial interests or personal relationships that could have appeared to influence the work reported in this paper.

## Data Availability

Data will be made available on request.

## Appendix Supplementary materials

Image, application 1
